# Supplementary material for: Patterns of Intron Gain and Loss in Fungi
Source: PLoS Biol. 2004 Nov 30;2(12):e422. doi: 10.1371/journal.pbio.0020422 (PMC532390; doi:10.1371/journal.pbio.0020422)
Supplement: Table S1 — Also available at http://genes.mit.edu/NielsenEtAl/. (4.3 MB ZIP). [file pbio.0020422.st001.zip › NielsenEtAl/html/24.html]

AN6547.1.NCU06712.1.MG04053.1.FG05222.1


```
 CLUSTAL W (1.82) Multiple Sequence Alignments - Introns Inserted


Sequence 1: NCU06712.1	265 aa
Sequence 2: MG04053.1	274 aa
Sequence 3: FG05222.1	254 aa
Sequence 4: AN6547.1	259 aa
Alignment Length: 274 aa
Number Identitical Residues: 155 aa
Alignment Score (without introns) 7134


MG04053.1 	MFRNNYDNDSVTF2SPQGRIFQVEYTAEAVKQGSVVVGLASKTHAILVAIK0RNAEELSS
NCU06712.1	MFRNNYDNDSVTF2SPQGRIFQVEYAAEAVKQGSVVVGIASKTHAVLVAIK0RNAEELSS
FG05222.1 	MFRNNYDNDSVTF2SPQGRIFQIEYAAEAVKQGSVVVGIASKTHAVLCAVK0RNAEELSS
AN6547.1  	MFRNNYDNDAVTF2SPQGRIFQVEYAQEAVKQGSVVVGLVNKTHAVLVGLK0RNAEELSS
          	*********:*** ********:**: ***********:..****:* .:* ********

MG04053.1 	YQKKLFPLDEHVGIAIAGLASDARVLSNFMKQQCLGHRLTYGRSMPVRTLVDLIGEKAQV
NCU06712.1	YQKKLFPIDEHVGIAIAGLTSDARVLSNFMKQQSLGHRLTYGRNMPVRTLVDMIGSKAQI
FG05222.1 	YQKKLFTVDEHAGIAIAGLTSDARVLSNFMKQQCLGHRLTYGRAIPLRSLVDMIGEKAQM
AN6547.1  	YQKKIIEVDSHMGVAIAGLASDARVLSNYMKQQCLGSRMTYGRPMPVNRIVSQIADRAQT
          	****:: :*.* *:*****:********:****.** *:**** :*:. :*. *..:** 

MG04053.1 	NTQHYGKRPYGVGLLVAGVDDRGPHLFDFQPSGMTEEMLAFAIGARSQMARTYLERNLDK
NCU06712.1	NTQHYGKRPYGVGLLVAGVDDHGPHLFEFQPSGMTEEMVAFAIGARSQMARTYLEKNLEA
FG05222.1 	NTQMYGKRPYGVGLLVAGVDERGPHLFEFQPSGMTEEMLAFAIGARSQMARTYLERNIDA
AN6547.1  	NTQQYGKRPYGVGLLVAGVDEAGPHLFEFQPSGMTQEMLACAIGARSQMARTYLERNLDK
          	*** ****************: *****:*******:**:* **************:*:: 

MG04053.1 	FADCDREELLKHGLRALKESLVQDKELTIENTSVGIVGLVKDGSGKSRVEPFRLYDGQEV
NCU06712.1	FADCGREELIRHGLKALKESLVQDRELTIDNTSVAIVGVEKQEDGTTKPQPFMVYDGQDV
FG05222.1 	FADCSKEELIQHGLKALKESLVQDKELSVENTSVGVVGIN-TVDGKKKVEPFKLYDGFSV
AN6547.1  	LQESSRDELITHGLRALKETLSQDKELTVDNTSVGVVGLA----DTGKVESFKLYEGQQL
          	: :..::**: ***:****:* **:**:::****.:**:     .. : :.* :*:* .:

MG04053.1 	KDWIDSVADDKEGGGDDSRPAAPAA~ASVSESMDVDE
NCU06712.1	SAWIESVADNQE--------AAPAA~AEG-EGMDVDS
FG05222.1 	QPWIESVGENQG--------GAEEA1-----------
AN6547.1  	LPLFEALDQSDA-----------SE~TKDEENMEVDS
          	   :::: :..               :. ... . ..
```
